# Global evaluation of the use of glycaemic impact measurements to food or nutrient intake

**DOI:** 10.1017/S1368980021000616

**Published:** 2021-08

**Authors:** Paula R Trumbo

**Affiliations:** EAS Consulting Group, LLC, 1700 Diagonal Road Suite 750, Alexandria, VA 22314, USA

**Keywords:** Glycaemic impact, Post-prandial glucose, Oral glucose tolerance test, Glycaemic index

## Abstract

**Objective:**

Measures of glycaemic impact (e.g. postprandial glucose (PPG), oral glucose tolerance test (OGTT) and glycaemic index (GI)) are used by government health and regulatory agencies and public health associations around the world. The objective of this global review was to identify similarities and differences in the use of glycaemic impact measures for potential considerations for harmonisation.

**Design:**

A literature and internet search was conducted to identify country government agencies and health associations that provide guidance or recommendations for PPG, OGTT, GI and glycaemic load.

**Results:**

Based on this global review, the use of GI for food labelling (e.g. low GI) is limited and its use is voluntary. The application of OGTT as a diagnostic measurement of diabetes and gestational diabetes is widely used and in a consistent manner among the different regions of the world. Time-specific (e.g. 2 h) PPG is commonly used as a target not to exceed in individuals with diabetes and gestational diabetes. PPG is used by regulatory agencies for the substantiation of food labelling. There are differences, however, among regulatory agencies in the specific measure of PPG (i.e. PPG AUC *v*. peak PPG). Maximum targets for 2-h PPG for individuals with diabetes and gestational diabetes, ranging between 6 and 10 mmol/l, across countries suggest a potential consideration to harmonise PPG targets.

**Conclusions:**

There is general consistency in the use and/or target levels of glycaemic impact measures; however, there is a potential need to investigate harmonisation strategies on certain aspects of glycaemic impact measures.

Glycaemic impact is the effect that a meal, food or nutrient has on short-term blood glucose levels after consumption. Glycaemic impact can be a function of the amount and type of carbohydrate (e.g. available carbohydrate and dietary fibre) consumed, the presence of other nutrients in a meal or food, food/ingredient form (e.g. solid *v*. liquid, raw starch *v*. cooked, intact grain *v*. flour) as well as the physiological state of the individual (e.g. type 2 diabetes)^(1)^. Available carbohydrate (also called glycaemic carbohydrate) is the carbohydrate that is digested and absorbed (e.g. starch and sugars) and provides a glycaemic impact. Carbohydrates that do not provide a glycaemic impact are often called non-glycaemic or non-digestible carbohydrates. There are several measures of glycaemic impact. Postprandial glucose (PPG) is a measurement of the concentration of glucose in plasma after consumption of a meal. In normal individuals, glucose levels peak at approximately 30–45 min after consumption of a meal^(2)^ and then return to preprandial levels within 2–3 h. In individuals with diabetes or healthy pregnant women, the peak is delayed to 1 h. The rise and fall of PPG levels are mediated by the first-phase insulin response, in which large amounts of endogenous insulin are released, usually within 10 min, in response to food intake^(3)^. The three general approaches to measuring PPG levels are (1) incremental AUC, (2) peak postprandial glucose (PkPPG) and (3) PPG measured at a certain time (e.g. 2 h) after consumption of a meal [e.g. 2-h PPG]). While PPG is measured in response to a meal, another short-term measurement of blood glucose, called oral glucose tolerance test (OGTT), is in response to a fixed amount of glucose. The OGTT is a surrogate meal that is designed to challenge glucose homoeostasis. It identifies individuals who are compromised and display levels that are dangerously high. Like PPG, there is a rise and fall in plasma glucose levels after consumption of glucose. PPG and OGTT are generally used to understand an individual’s glycaemic impact on consumption of available carbohydrate. Whereas another measure of glycaemic impact, glycaemic index (GI), is designed to rank the glycaemic impact of individual foods, but not mixed meals^(4)^. GI is unitless and is a relative ranking of carbohydrate in foods according to how they affect blood glucose levels^(5)^. The GI of a food is determined based on its relative glycaemic impact on 50 g of digestible (available) carbohydrate. Glycaemic load (GL) is derived in a similar way; however, unlike GI, GL is an indicator of the relative glycaemic impact of a given amount of carbohydrate in the individual food^(2)^. The relative GL of meals and whole diets can be calculated.

The above-described measures of glycaemic impact can have different uses by governments, such as regulatory agencies (e.g. authoritative agencies), international health and public health associations. This paper examines the working definition and measurement of available carbohydrate, and based on a global review, examines the use of the glycaemic impact measurements across different regions of the world and provides an overall summary of the similarities and differences of each measurement’s application, including food labelling, dietary guidance and clinical recommendations. From these findings, an objective is to determine whether there is a need to harmonise the use of and/or target levels for different measures of glycaemic impact.

## Methods and results

For this global review, Yahoo, Google and PubMed were used to identify government regulatory and health agencies, relevant public health associations (e.g. diabetes associations) and international organisations and associations (e.g. WHO). This is a narrative review using a systematic approach. Search terms included within each identified website and for PubMed were ‘post prandial glucose,’ ‘oral glucose tolerance test,’ ‘glycemic index,’ ‘glycaemic index,’ ‘glycemic load’ and ‘glycaemic load.’ Using these search terms also identified information on a part of the term (e.g. ‘index’). To ensure that the findings of the short-term measurements were globally represented, major food markets of the top five countries per regions (Americas, Europe, Oceana, Asia and the Middle East) were identified, based on the total production Index of FAO representing net food production normalised to international index^(6)^. Additional countries were included if it was known that a government agency or health association provided relevant information on glycaemic impact measures. Assistance was requested by International Life Sciences Institute (ILSI) regional/branch representatives in identifying individuals or seeking information related to the application of glycaemic impact measurements in different countries.

Based on this global review, tables (see online supplementary material, Supplemental Tables 1–4) were developed that identify the countries included in the review and relevant information from their government agencies and health associations, along with their website address. Information was considered to be not available if (1) a relevant website was not found, (2) the website did not provide the ability to search for relevant information, (3) the website search did not identify any relevant information or (4) the website and/or information was not in English. Information from the supplemental tables was used as the basis for the discussion on the glycaemic impact measurement discussed below. Supplemental Tables 1–4 also provide additional information to what is discussed in this review.

### Postprandial glucose levels

In measuring postprandial glucose AUC (PPG AUC), plasma glucose levels are measured immediately before and multiple times after consumption of a meal. The rise and fall of plasma glucose levels yield a curve under which an area can be calculated such that a typical unit of measure is mmol × min/l or mg × min/d^(7,8)^ (Table 1). Peak postprandial glucose (PkPPG) is the highest concentration of plasma glucose that is measured after consumption of a meal. PkPPG and PPG measured at a certain time after a meal (e.g. 2 h) are often report as mg/dl or mmol/l (Table 1).

**Table 1:** Calculation of glycaemic impact and units of measurement

Measurement	Calculation	Unit of measure
PkPPG	Highest BG concentration	mg/dl mmol/l
2-h PPG	BG concentration at 2 h after consumption of a meal	mg/dl mmol/l
PPG AUC	Trapezoidal approximation^(56)^	mg · min/dl mmol · min/l
2-h OGTT	BG concentration 2 h after consumption of 75 g glucose	mg/dl mmol/l
Glycaemic Index of a food	(AUC_ReferenceFood_ ÷ AUC _StandardFood)_ × 100 AUCs are determined by trapezoidal approximation^(56)^ Reference food is tested two to three times	
Glycaemic Index of a meal	(GI meal = GI_FoodA_ × Available Carb_FoodA_ + GI_Food B_ × g Available Carb_FoodB_ +………………………….) ÷ Total Available Carb	
Glycaemic load	(GI × carbohydrate content per portion) ÷ 100	g per serving g per 100 g food g per 1000 kJ or 1000 kcal

PkPPG, peak postprandial glucose; BG, blood glucose; GI, glycaemic index; Carb, carbohydrate; AUC, area-under-the curve.

### Postprandial glucose and food labelling

Five authoritative organisations were identified that use PPG AUC and/or PkPPG for making science-based regulatory decisions for food labelling.

The US FDA considers a reduction in PPG AUC and/or PkPPG to be physiological effects that are beneficial in humans who consume specific dietary fibres^(9)^. Studies are required to show that when an isolated or synthetic nondigestible carbohydrate (NDC) is added to a food or beverage, the PPG AUC and/or PkPPG is significantly reduced (*P* ≤ 0·05) compared with a control food or beverage that does not contain the NDC. Evidence that demonstrates that a specific isolated or synthetic NDC attenuates PPG AUC and/or PkPPG can be used to support the NDC in meeting FDA’s definition of dietary fibre which allows the amount of that NDC ingredient to be declared as dietary fibre on the Nutrition Facts label^(10)^. The amount of available carbohydrate should be the same between the treatment and control diet. Otherwise, it is not possible to determine if there is an independent effect of the NDC or if the effect is due to differences in amounts of available carbohydrate being consumed, which would be expected to impact blood glucose concentrations. As an alternate example, for meeting the FDA definition of dietary fibre, FDA used reduced post-prandial insulin response in the absence of a rise on PPG AUC as a beneficial physiological effect of resistant starch 2 because less insulin is required to achieve a similar glycaemic effect^(11)^. FDA does not consider PPG AUC or PkPPG per se to substantiate health claims because such claims pertain to disease risk and PPG is not considered a surrogate endpoint for risk of chronic disease, such as type 2 diabetes^(12)^.

In Canada, for the labelling of foods with function claims related to the reduction of glycaemic impact, the primary outcome considered is PPG AUC over at least a 2-h period. Health Canada considers a minimum 20 % decrease in the average incremental AUC (ignoring the area beneath the fasting concentration which is how AUC is usually measured) in comparison with the reference food to be a physiologically relevant reduction^(13)^. This magnitude of change must also be statistically significant. Health Canada does not consider PkPPG to be sufficient to measure glycaemic impact, but can be used as supportive data, when correlating with PPG AUC.

The European Food Safety Authority (EFSA) considers PPG AUC and PkPPG to be appropriate as primary endpoints to substantiate health claims regarding the reduction in PPG, whereas it can be used only as supportive evidence to substantiate health claims in the context of (long-term) maintenance of normal glucose regulation^(14)^. EFSA has noted, however, that PPG AUC and PkPPG should be used in combination with insulin AUC to exclude a disproportionate increase in insulin values in comparison with the control food/meal.

For function claims, the Food Standards Australia New Zealand (FSANZ) has considered PkPPG, but not PPG AUC, when reviewing the effect of dietary fibres, such as pectin and beta-glucan, on postprandial glycaemic impact^(15)^. FSANZ considers PkPPG the most appropriate measure because it is the most uniformly reported measurement and also measures immediate postprandial effect.

### Postprandial glucose guidelines and targets

Various guidelines and targets on PPG from authoritative organisations and health associations around the world were identified. Targets were specific for pregnant women, pregnant women with gestational diabetes and individuals with hyperglycaemia or type 2 diabetes. Rather than using PPG AUC or PkPPG, most of the targets are based on PPG at a fixed time after consumption.

The Fiji Ministry of Health and Medical Services recommends a 2-h PPG target of 5–7 mmol/l glucose during pregnancy^(16)^. New Zealand Ministry of Health and India’s Ministry of Health and Family Welfare have identified a 2-h PPG level of < 6·5 mmol/l to achieve in women with gestational diabetes^(17,18)^.

The American Diabetes Association (ADA) recommends postprandial self-monitoring of blood glucose in both gestational diabetes mellitus and preexisting diabetes in pregnancy to achieve glycaemic control. During pregnancy, reducing 1- and 2-h PPG levels to < 10 mmol/l may help, in part, to lower HbA1C in individuals with type 2 diabetes^(19)^. For pregnant women with gestational diabetes, the 1-h cut-off is lower at < 7·8 mmol/l (2-h PPG cut-off is < 6·5 mmol/l)^(19)^.

The US Center for Disease Control and Prevention (CDC) provides a target of < 10 mmol/l for all Americans with diabetes^(20)^. Likewise, targets for individuals with type 2 diabetes have been provided by several associations around the world. The European Society of Cardiology/European Association for the Study of Diabetes recommends a PkPPG of no more than 7·5 mmol/l in individuals with type 2 diabetes^(21)^. For people with type 2 diabetes, Diabetes UK recommends a 2-h PPG < 8·3 mmol/l^(22)^, Diabetes Australia provides a 2-h PPG target of 6–10 mmol/l^(23)^ and the Diabetic Association of Pakistan^(24)^ and the Brazil Diabetes Society^(25)^ recommend a 2-h PPG target of < 0·9 mmol/l. The International Diabetes Foundation (IDF) provides guidelines that recommend a 2-h PPG target of < 7·8 mmol/l for individuals with hyperglycaemia^(26)^. The IDF has noted that glucose levels in healthy people are often difficult to achieve in people with diabetes without an undue risk of hypoglycaemia. Therefore, for reasons of safety, the IDF provides a PPG target of 8·8 mmol/l^(27)^. It is not clear at what time point after consumption this level pertains to.

### Postprandial glucose and diagnosis of type 2 diabetes

The WHO provides various diagnostic biomarker cut-offs for type 2 diabetes^(28)^. WHO has defined a person who has diabetes as having a 2-h PPG level above 11 mmol/l on two separate occasions. No other organisations were identified that used PPG as a diagnostic marker for type 2 diabetes.

### Oral glucose tolerance test

Standard protocol for conducting an OGTT involves drawing a fasting venous blood sample before administering 75 g glucose. Blood is then drawn again 2 h after glucose intake^(29)^. Serum or plasma glucose can be measured. Typical units of measurement are mg/dl or mmol/l (Table 1).

### Oral glucose tolerance test and food labelling

Two authoritative organisations were identified in which OGTT data are used in the scientific substantiation of claims for food labelling. The US FDA considers OGTT to be one of several surrogate biomarkers of type 2 diabetes risk^(12)^. As such, OGTT data are used to substantiate a health claim for a relationship between a food or food component and type 2 diabetes risk. Because OGTT is also a diagnostic biomarker of type 2 diabetes, OGTT is also used by the US FDA to identify studies that are and are not conducted on individuals with type 2 diabetes. Studies that are conducted on individual with type 2 diabetes generally are not used to substantiate a health claim which is about risk reduction, rather than treatment.

EFSA uses OGTT data to substantiate health claims for glucose tolerance^(30)^. The scientific evidence for the substantiation of health claims related to an increase in glucose tolerance is obtained from human intervention studies showing a decrease in blood glucose concentrations at different time points during an OGTT and with no disproportionate increase in insulin concentrations following chronic consumption (at least 12 weeks) of the food that is the subject of the health claim.

### Oral glucose tolerance tests and diagnosis of diabetes

The US National Institutes of Health (NIH) considers a 2-h OGTT of > 11 mmol/l as indicative of diabetes^(31)^. These 2-h blood levels for prediabetes and diabetes are recognised by international organisations such as the WHO/IDF^(32)^. The WHO/IDF has stated that OGTT is the only means to identify people with impaired glucose tolerance^(32)^. The WHO, including the Pan American Health Organization, considers a 2-h blood glucose range of 7·8–11 mmol/l to be a risk factor for types 2 diabetes when other risk factors exist^(33)^. Public Health Canada uses OGTT for monitoring of dysglycaemia (> 7·8 mmol/l)^(34)^.

In addition to fasting venous blood glucose, various diabetes associations throughout the world consider OGTT to be an important diagnostic biomarker of prediabetes and diabetes. A range for a 2-h blood glucose level of 7·8–11 mmol/l is indicative of prediabetes or impaired glucose tolerance, and/or a 2-h blood glucose level of ≥ 11·1 mmol/l is indicative of type 2 diabetes by various diabetes associations in the Brazil, United States, Canada, United Kingdom, Japan, China, New Zealand and India^(25, 35–41)^.

The Singapore Ministry of Health recommends that, for women with gestational diabetes, an OGTT be performed 6–12 weeks postpartum and the woman reclassified and counselled according to criteria accepted in the non-pregnant state^(42)^. Oral Glucose Tolerance Tests have been used in the national screening of the prevalence of diabetes. In 2015–2016, as well as some prior years, the US National Health and Nutrition Examination Survey measured OGTT^(43)^.

Some associations, such as the American Diabetes Association^(44)^, Diabetes South Africa^(45)^ and the Japanese Diabetes Society^(38)^, have identified 2-h blood glucose levels of greater than 8·6 mmol/l for the diagnosis of gestational diabetes. Besides associations, government agencies such as the Fiji Ministry of Health use 8·5 mmol/l as a cut-off for the diagnosis of gestational diabetes^(16)^. Because of the risk of gestational diabetes during pregnancy, associations, such as Diabetes New Zealand, recommend that all pregnant women should have an OGTT conducted at 28 weeks^(40)^, whereas the Diabetes Association of Nigeria recommends the performance of an OGTT in all at-risk pregnant women^(46)^. Diabetes Australia recommends that an OGTT be performed six weeks after delivery to ensure that blood glucose levels have returned to normal^(47)^. The International Association for the Study of Diabetes in Pregnancy has attempted to harmonise diagnostic cut-off levels^(48)^.

### Glycaemic Index

The glycaemic index (or GI) is a ranking of carbohydrates on a scale from 0 to 100 according to the extent to which they raise blood sugar (glucose) levels after eating. Foods with a high GI are those which are rapidly digested and absorbed^(49)^. Unlike PPG and OGTT in which blood glucose levels provide information about how an individual handles the consumption of available carbohydrates, GI is used to compare foods. The GI of mixed meals can be calculated and ranked using weighted means of individual foods (Table 1). It is a property of the food itself and is an index or percentage representing a quality of carbohydrate-containing foods^(49)^. The GI of a food can fall into one of three categories: low < 55, medium 55–69 and high > 70^(49)^.

Measurement of GI requires testing of individual foods. Following fasting, 10 or more healthy people consume a portion of the test food containing 50 g of digestible (available) carbohydrate and then measuring the effect on their blood glucose levels over the next 2 h. For each person, the area under their 2-h blood glucose response (glucose AUC) for this food is then measured^(7,8)^. On at least two or more occasions, the same 10 people consume an equal carbohydrate portion of the sugar glucose (the reference food) and their 2-h blood glucose response is also measured^(49)^. The AUC of the test food is divided by the AUC of the reference (either glucose or white bread providing 50 g of available carbohydrate) and multiplied by 100^(50)^ (Table 1). It is critical to test the reference foods two to three times to help reduce the se around the mean value. Health Canada has noted that measurements should be taken for at least 2 h, with higher frequency (for example, at 15-min intervals) in the first hour and 30 min thereafter^(13)^. The insulin response to a food should be proportional to the post-prandial glycaemic impact. Therefore, data on insulin concentrations following the consumption of the test food should be provided to show that the decrease in blood glucose concentrations is not accompanied by disproportionately increased levels of insulin, in comparison with the reference food^(13)^.

The GI of mixed meals is calculated from the GI of the carbohydrate of foods or ingredients in the meal, rather than directly^(4)^. The GI of individual foods, weighted according to the amount of carbohydrate each food contributes to the meal, has been devised to estimate the GI of whole meals (Table 1). A problem with the use of published values is that the GI is affected by factors such as macronutrient content, variety, ripeness, processing and cooking. To overcome this problem, the direct calculation of the GI of individual foods is recommended^(51)^.

### Glycaemic index, food labelling and recommended intake levels

Some regulatory agencies identified in this review do not use GI for the purposes of food labelling. The US FDA addressed the fact that GI does not measure physiological benefits of nutrients added to foods such as dietary fibre^(9)^. Therefore, evidence on GI is not used for considering if a non-digestible carbohydrate meets the FDA definition of dietary fibre for declaration in the Nutrition Facts label. Health Canada has stated that inclusion of the GI value on the label of eligible food products would be misleading and would not add value to nutrition labelling and dietary guidelines in assisting consumers to make healthier food choices^(52)^.

Current South African labelling regulations permit GI and GL labelling related to health claims^(53)^. Voluntary GI information can be provided on labels by way of the Glycemic Index Foundation of South Africa (GIFSA) endorsement logo which has been accredited by the South African Department of Health, as the GI testing done by them is in accordance with international standards and must meet other criteria^(54)^.

The FSANZ allows companies to make nutrient content claims regarding the GI of a food^(55)^. The voluntary certified GI symbol licensed by the Glycemic Index Foundation indicates the GI rating of packaged food products in supermarkets^(56)^. It ranks food products based on the speed at which they break down from carbohydrate to sugar in the bloodstream. However, this labelling is not required for food companies to follow. The GI symbol may appear only on food products that meet certain nutrient criteria for that food category^(57)^. High and intermediate GI soft drinks, cordials, syrups, confectionery and sugars are excluded. Jams, honey and other carbohydrate-containing spreads are not necessarily excluded. Nutrition content claims can be made about GI, but the food must meet the government’s nutrient profiling score criterion. When making a claim about GI, the specific numerical value of the GI of the food must be included either in the claim or in the nutrition information panel. The descriptors low, medium and high are optional in a GI claim, but if used, must meet certain conditions. The GI symbol is a front-of-pack labelling scheme that requires a GI value in the Nutrition Facts/Nutrition Information Panel. GI function claims on satiety, sustained energy and physical performance are permitted via a notification to FSANZ^(58)^. The foods must meet various criteria and have scientific substantiation on file. The Commonwealth Scientific and Industrial Research Organisation, an Australian government industrial research organisation, partners with the Glycemic Index Foundation to provide the GI symbol program with specified product eligibility and nutrient criteria^(59)^.

Singapore’s Health Promotion Board allows for a ‘Low Glycaemic Index’ claim if a food has a GI value of 55 or less^(60)^. Likewise, the Food Safety and Standards Authority of India permits a ‘Low GI’ claim when the GI value is < 55^(61)^.

The WHO/FAO has stated that the choice of carbohydrate-containing foods should not be based solely on GI since low-GI foods may be energy dense and contain substantial amounts of sugars, fat or undesirable fatty acids that contribute to the diminished glycaemic impact, but not necessarily to good health outcomes^(62)^. GI is perhaps most appropriately used to guide food choices when considering similar carbohydrate-containing foods^(62)^. GI should always be considered in the context of other nutritional indicators^(62)^. While foods with a low GI may also confer benefit in some of these contexts, FAO/WHO further notes that the scientific evidence suggests caution regarding the use of the GI as the sole determinant of the quality of carbohydrate-containing foods. The FAO has also stated that GI can be used, in conjunction with information about food composition, to guide food choices. For practical application, the GI is useful to rank foods by developing exchange lists of categories of low GI foods, such as legumes, pearled barley, lightly refined grains (e.g. whole grain pumpernickel bread, or breads made from coarse flour) and pasta^(62)^.

In a scientific opinion on dietary reference values for carbohydrates, EFSA stated that a cause–effect relation could not be established between low-GI carbohydrate foods and claimed functional effects^(63)^. EFSA noted that, although there is some experimental evidence that a reduction of the dietary GI may have favorable effects on some metabolic risk factors such as serum lipids, the evidence for a role in weight maintenance and prevention of diet-related diseases is inconclusive. The German Nutrition Society issued a document on dietary reference values in which it was concluded that there is only possible evidence regarding a risk-increasing effect of high GI on some nutrition-related diseases^(64)^. Therefore, no recommendations were made with respect to GI.

### Glycaemic index and dietary guidance

A number of government agencies and associations have reviewed that scientific evidence and/or have provided some form of dietary guidance related to GI. Public Health England’s Scientific Advisory Committee on Nutrition conducted a science review on carbohydrates and health. It was concluded that it is not possible to assign cause–effect relationships for outcomes based on variation in dietary GI, as higher or lower GI diets differ in many ways other than just the carbohydrate fraction^(65)^. Fiji Ministry of Health and Medical Services recommends that individuals with diabetes aim to consume foods with a low GI^(16)^. Instead of dietary guidance for people with diabetes, the NIH has provided recommendations for GI to individuals with nonalcoholic fatty liver disease or nonalcoholic steatohepatitis^(66)^. The NIH recommends that such individuals eat more low GI foods, such as most fruits, vegetables and whole grains.

While recommendations are not provided, based on the American Diabetes Association evidence grading system for clinical practice recommendations, it was concluded that there was supporting evidence from observational studies that the use of the glycaemic index and glycaemic load may provide a modest additional benefit for glycaemic control over that observed when total carbohydrate is considered alone^(67)^. Diabetes Australia recommends to eat more low and intermediate GI foods, but not to exclude high GI foods^(68)^. For individuals with pre-diabetes or diabetes, Diabetes Canada recommends choosing lower GI foods and drinks more often to help control blood sugar^(69)^. Diabetes Canada’s most recent education materials have been designed to support healthcare providers and people affected by diabetes as they learn about GI together. South Africa’s Food Advisory Consumer Service has noted that a GI value can assist in selecting foods that is high in fibre, micronutrients and antioxidants and low in energy, which is the basis of a healthy diet^(70)^.

Diabetes United Kingdom’s Guide to Diabetes states that research has shown that choosing low GI foods can particularly help manage glucose levels in people with Type 2 diabetes^(71)^. There is less evidence to suggest it can help with blood glucose control in people with Type 1 diabetes. However, the guide notes that not all low-GI foods are healthy choices; chocolate, for example, has a low GI, but has a high fat content. Eating to control diabetes is not just about GI ratings, but also about consuming foods low in saturated fat and salt as part of a healthy, balanced diet. Combining foods with different GIs alters the overall GI of a meal, and the benefit of GI can be maximised by switching to a low GI option with each meal or snack.

### Glycaemic load

The GL is the product of GI and the total carbohydrate content in a given amount of food (GL = GI × carbohydrate/given amount of food)^(72)^ (Table 1). The relative GL of meals and whole diets can be calculated based on the GL of an individual foods. GL has corresponding units of g per serving, g per 100 g food and g per 1000 kJ or 1000 kcal. The GL of a mixed meal or diet can simply be calculated by summing together the GL values for each ingredient or food component^(72)^. Like GI, there are three categories of GL: low 10 or less, medium 11–19, high 20 or more^(72)^.

### Glycaemic load and recommendations

In South Africa, a GL health claim is permissible, in part, if the GI category is indicated as well^(53)^. Several authoritative agencies and organisations have commented on GL. EFSA has stated that the evidence for a role of GL in weight maintenance and prevention of diet-related diseases is inconclusive^(63)^. Public Health England’s Scientific Advisory Committee on Nutrition concluded that it was not possible to identify cause–effect relationships for outcomes based on variation in diet GL, as higher or lower GL diets differ in many ways other than just the carbohydrate fraction^(65)^. WHO/FAO has stated that GL should always be considered in the context of other nutritional indicators^(62)^. FSANZ permits nutrient content claims about GL; however, the food must meet certain nutrition profiling score criterion^(55)^. The Indian Council of Medical Research recommends that individuals with type 2 diabetes should consume carbohydrates from foods that are high in fibre (e.g. whole grains, legumes, peas, beans, oats, barley and some fruits) with a low GL^(41)^. The Glycemic Index Foundation has stated that although the GL concept has been useful in scientific research, it is the GI that has proven most helpful to people with diabetes and those who are overweight^(72)^.

## Conclusions

Based on the global review of the use of glycaemic impact measurements, it can be seen that different measures are used, but when comparing specific measures, the cut-off levels are generally consistent. The application of post-prandial glucose response is relevant particularly within the context of available carbohydrate and certain dietary fibres. For those identified regulatory agencies that conduct pre-market scientific reviews for food labelling, PPG, as a physiological endpoint, is used for function claims on glycaemic impact or in evaluating the beneficial physiological effects of isolated or synthetic NDCs for being declared as a dietary fibre in the US Nutrition Facts label (Table 2). As previously discussed, there were some differences in the use of PPG AUC and PkPPG. While the target level varies across authoritative agencies and public health organisations in individuals with diabetes (< 6–10 nmol/l) and gestational diabetes (< 6·5–10 mmol/l), time-specific (e.g. 2 h) PPG is commonly used as a target not to exceed. An objective of this global review was to determine whether there is a need to harmonise the use of and target levels for different measures of glycaemic impact. A potential area for consideration is the harmonisation in the use of PPG AUC and PkPPG in scientific regulatory reviews. Are there certain situations in which PPG AUC is more appropriate to use than PkPPG and visa versa? When can both be considered? Is there a fundamental reason why regulatory agencies would treat PPG AUC and PkPPG differently? Maximum targets for 2-h PPG for individuals with diabetes and gestational diabetes, ranging between 6 and 10 mmol/l, across countries also suggests a potential consideration to harmonise PPG targets.

**Table 2:** Global use of glycaemic impact measurements

	Food Labelling	Targets	Diagnosis
PPG	Substantiation of functional claims Substantiation of the physiological effects of dietary fibre that is required for declaration of the amount in the food label	2-h Diabetes (< 6 to 10 mmol/l) Gestational diabetes (< 6·5–10 mmol/l)	Uncommon
OGTT	Substantiation of health claims on type 2 diabetes/glucose tolerance	Uncommon	Prediabetes (7·8–11 mmol/l) Diabetes (≥ 11·1 mmol/l) Gestational diabetes (≥ 8·5 mmol/L)
GI	Front-of-package claims Nutrient-content claims	Diabetes “Low GI” (< 0 50)	No

PPG, post-prandial glucose; OGTT, oral glucose tolerance test; GI, glycaemic index.

The application of OGTT as a diagnostic measurement of diabetes and gestational diabetes is widely used and in a consistent manner among the different regions of the world (Table 2). A few authoritative organisations use OGTT as a biomarker of type 2 diabetes risk/glucose tolerance. An OGTT level of ≥ 11·1 mmol/l is used to diagnose diabetes; the level for gestational diabetes is generally ≥ 8·5 mmol/l (Table 2). As such, there is general harmonisation in the use of OGTT and OGTT targets.

Based on the countries identified in this review, the use of GI for labelling is limited with the greatest use being in Australia, New Zealand and South Africa. Some government agencies and health associations have expressed views regarding the limitations in using GI for making policy decisions and health recommendations. Where labelling of GI is present, it is voluntary rather than mandatory. When GI is used, however, there is general harmonisation in the levels used to categorise the GI of a food (e.g. low).

## Supplementary material

For supplementary material accompanying this paper visit https://doi.org/10.1017/S1368980021000616. click here to view supplementary material
