# Supplementary material for: Global evaluation of the use of glycaemic impact measurements to food or nutrient intake
Source: Public Health Nutr. 2021 Feb 10;24(12):3966–75. doi: 10.1017/S1368980021000616 (PMC8369455; doi:10.1017/S1368980021000616)
Supplement: Supplementary file 1 [file S1368980021000616sup.zip › S1368980021000616sup001.docx]

**References for Supplemental Tables 1-4**

- - - 1. Health Canada (2016) Summary of Health Canada's assessment of a health claim about a polysaccharide complex (glucomannan, xanthan gum, sodium alginate) and a reduction of the post-prandial blood glucose response. <https://www.canada.ca/en/health-canada/services/food-nutrition/food-labelling/health-claims/assessments/summary-assessment-health-claim-about-polysaccharide-complex-glucomannan-xanthan-sodium-alginate-reduction-post-prandial-blood-glucose.html> (accessed June 2020).
      2. Health Canada (2013) Draft guidance document on Food Health Claims related to post-prandial glycaemia. <https://www.canada.ca/en/health-canada/services/food-nutrition/public-involvement-partnerships/technical-consultation-draft-guidance-document-food-health-claims-related-post-prandial-glycaemia.html> (accessed May 2020).
      3. Health Canada (2017) List of Dietary Fibres Reviewed and Accepted by Health Canada’s Food Directorate. <https://www.canada.ca/en/health-canada/services/publications/food-nutrition/list-reviewed-accepted-dietary-fibres.html> (accessed June 2020).
      4. US Food and Drug Administration (2018) Scientific Evaluation of the Evidence on the Beneficial Physiological Effects of Isolated or Synthetic Non-Digestible Carbohydrates Submitted as a Citizen Petition (21 CFR 10.30): Guidance for Industry. <https://www.fda.gov/media/101183/download> (accessed May 2020).
      5. US Food and Drug Administration (2018). The Declaration of Certain Isolated or Synthetic Non-Digestible Carbohydrates as Dietary Fiber on Nutrition and Supplement Facts Labels: Guidance for Industry. <https://www.fda.gov/media/113663/download> (accessed May 2020).
      6. European Food Safety Authority (2010) Scientific Opinion on Dietary Reference Values for carbohydrates and dietary fibre. *EFSA Journal* **8**, 1462.
      7. Food Standards New Zealand (2016) Systematic review of the evidence for a relationship between pectin and peak postprandial blood glucose concentration. <https://www.foodstandards.gov.au/consumer/labelling/nutrition/Documents/Pectin%20glucose.pdf> (accessed May 2020).
      8. International Diabetes Federation (2013) 2011 Guideline for management of postmeal glucose in diabetes.<https://www.idf.org/e-library/guidelines/82-management-of-postmeal-glucose.html> (accessed May 2020).
      9. [de Almeida-Pititto](https://www.ncbi.nlm.nih.gov/pubmed/?term=de%20Almeida-Pititto%20B%5BAuthor%5D&cauthor=true&cauthor_uid=25609989) B,  [Dias](https://www.ncbi.nlm.nih.gov/pubmed/?term=Dias%20ML%5BAuthor%5D&cauthor=true&cauthor_uid=25609989) ML, [Franco de Moraes](https://www.ncbi.nlm.nih.gov/pubmed/?term=de%20Moraes%20AC%5BAuthor%5D&cauthor=true&cauthor_uid=25609989) AC et al. (2015) Type 2 diabetes in Brazil: epidemiology and management. [Diabetes *Metab Syndr Obes*](https://www.ncbi.nlm.nih.gov/pmc/articles/PMC4298341/). **8**, 17–28.
      10. Center for Disease Control and Prevention (2019) Manage Blood Sugar. <https://www.cdc.gov/diabetes/managing/manage-blood-sugar.html> (accessed May 2020).
      11. American Diabetes Association. The big picture: checking your blood glucose. <https://www.diabetes.org/diabetes/medication-management/blood-glucose-testing-and-control/checking-your-blood-glucose> (accessed June 2020).
      12. American Diabetes Association (2019) Management of Diabetes in Pregnancy: Standards of medical care in diabetes – 2019. *Diabetes Care* **42**, S165-S172.
      13. American Diabetes Association (2018) Glycemic targets: Standards of medical care – 2018. *Diabetes Care* **41**, S55-S64.
      14. American Diabetes Association (2014) Standards of medical care in diabetes – 20014. *Diabetes Care* **37**, S14-S80.
      15. Martini D, Biasini B, Zavaroni I et al. (2018) Claimed effects, outcome variables and methods of measurement for health claims proposed under European Community Regulation 1924/2006 in the area of blood glucose and insulin concentrations*. Acta Diabetologica* **55**, 391–404.
      16. The Task Force on Diabetes and Cardiovascular Diseases of the European Society of Cardiology (ESC) and of the European Association for the Study of Diabetes (EASD) (2007) Guidelines on diabetes, pre-diabetes, and cardiovascular diseases: executive summary. European Heart Journal **28**, 88–136.
      17. Diabetes UK. Guide to Diabetes: Checking your blood sugar levels. <https://www.diabetes.org.uk/guide-to-diabetes/managing-your-diabetes/testing> (accessed May 2020).
      18. Food Standards Australia New Zealand (2016) Systematic review of the evidence for a relationship between pectin and peak postprandial blood glucose concentration. <https://www.foodstandards.gov.au/consumer/labelling/nutrition/Documents/Pectin%20glucose.pdf> (accessed May 2020).
      19. New Zealand Ministry of Health (2014) Diabetes in Pregnancy: Quick reference guide for health professionals on the screening, diagnosis and treatment of gestational diabetes in New Zealand. <https://www.health.govt.nz/system/files/documents/publications/diabetes-in-pregnancy-quick-reference-guide-dec14-v4.pdf> (accessed May 2020).
      20. Diabetes Australia. Living with diabetes: Blood glucose monitoring. <https://www.diabetesaustralia.com.au/blood-glucose-monitoring> (accessed May 2020).
      21. Fiji Ministry of Health (2012) Diabetes Management Guidelines. <http://www.health.gov.fj/wp-content/uploads/2018/03/Diabetes-Management-Guidelines.pdf> (accessed May 2020).
      22. India Ministry of Health and Family Welfare (2018) Diagnosis & Management of Gestational Diabetes Mellitus. Technical and Operational Guidelines. <https://nhm.gov.in/New_Updates_2018/NHM_Components/RMNCH_MH_Guidelines/Gestational-Diabetes-Mellitus.pdf> (accessed May 2020).
      23. Diabetes Association of Pakistan. About Diabetes: Managing your diabetes. <http://www.dap.org.pk/AbouDiabetes.html> (accessed May 2020).
      24. Diabetes South Africa (2017) Diagnosis Diabetes. <https://www.diabetessa.org.za/frequently-asked-questions/> (accessed June 2020).
      25. Ceriello A & Colagiuri S. (2008) International Diabetes Federation guideline for management of postmeal glucose: a review of recommendations. *Diabet Med*. 25, 1151–1156.
      26. International Diabetes Federation (2013) 2011 Guideline for management of postmeal glucose in diabetes. <https://www.idf.org/e-library/guidelines/82-management-of-postmeal-glucose.html> (accessed May 2020).
      27. World Health Organization. WHO/ISH risk prediction charts. <https://www.who.int/ncds/management/WHO_ISH_Risk_Prediction_Charts.pdf?ua=1> (accessed May 2020).
      28. World Health Organization (2007) Prevention of Cardiovascular Disease Guidelines for assessment and management of cardiovascular risk. <https://ish-world.com/downloads/activities/71665_71665_OMS_INT-RETIRATION.pdf> (accessed May 2020).
      29. Agarwal G, Jiang Y, Van Katwyk SR et al. (2018) Effectiveness of the CANRISK tool in the identification of dysglycemia in First Nations and Métis in Canada. *Health Promotion and Chronic Disease Prevention in Canada* **38**, 55-63.
      30. Canadian Diabetes Association (2013). Canadian Diabetes Association Clinical Practice Guidelines.<http://www.gov.pe.ca/photos/original/hpei_diabguidel.pdf> (accessed May 2020).
      31. Canadian Diabetes Association (2013). Gestational diabetes and postpartum screening. <https://www.diabetes.ca/DiabetesCanadaWebsite/media/Managing-My-Diabetes/Tools%20and%20Resources/gestational-diabetes-postpartum-screening.pdf?ext=.pdf> (accessed May 2020).
      32. US Food and Drug Administration (2009). Guidance for Industry: Evidence-Based Review System for the Scientific Evaluation of Health Claims. <https://www.fda.gov/regulatory-information/search-fda-guidance-documents/guidance-industry-evidence-based-review-system-scientific-evaluation-health-claims> (accessed May 2020).
      33. National Institute of Diabetes and Digestive and Kidney Disease (2014). Diabetes and Prediabetes Tests. <https://www.niddk.nih.gov/health-information/professionals/clinical-tools-patient-management/diabetes/diabetes-prediabetes> (accessed May 2020).
      34. Center for Disease Control and Prevention. National Health and Nutrition Examination Survey. 2015-2016 Data Documentation, Codebook, and Frequencies. Oral Glucose Tolerance Test (OGTT_I). <https://wwwn.cdc.gov/Nchs/Nhanes/2015-2016/OGTT_I.htm> (accessed May 2020).
      35. American Diabetes Association. Diagnosis. <https://www.diabetes.org/a1c/diagnosis> (accessed May 2020).
      36. American Diabetes Association (2010) Position Statement: Diagnosis and classification of diabetes mellitus. *Diabetes Care* **33**, S62-S69.
      37. European Food Safety Authority (EFSA) Panel on Dietetic Products, Nutrition and Allergies (2014) Scientific Opinion on the substantiation of a health claim related to olive (Olea europaea L.) leaf water extract and increase in glucose tolerance pursuant to Article 13(5) of Regulation (EC) No 1924/20061. *EFSA Journal* **12**, 3655.
      38. Diabetes UK (2019) Diabetes Care: Postprandial plasma glucose test. <https://www.diabetes.co.uk/diabetes_care/postprandial-plasma-glucose-test.html> (accessed May 2020).
      39. Australian Government Department of Health. Health Direct (2018) Diabetes Diagnosis. <https://www.healthdirect.gov.au/diabetes-diagnosis> (accessed May 2020).
      40. Food Standards Australia New Zealand (2016) Nutrition, Health and related claims. <http://www.foodstandards.gov.au/industry/labelling/Pages/Nutrition-health-and-related-claims.aspx> (accessed May 2020).
      41. Diabetes Australia. Managing gestational diabetes. <https://www.diabetesaustralia.com.au/managing-gestational-diabetes> (accessed May 2020).
      42. Diabetes New Zealand. What Tests Can be Done To Find Out If I Have Diabetes? <https://www.diabetes.org.nz/diabetes-diagnosis?rq=tolerance> (accessed May 2020).
      43. [Jia](https://onlinelibrary.wiley.com/action/doSearch?ContribAuthorStored=Jia%2C+Weiping) W, Weng J, Zhu D et al. (2019) Standards of Medical Care for Type 2 Diabetes in China. *Diabetes Metab Res Rev*. **35**:e3158.
      44. Indian Council of Medical Research (2018). ICMR Guidelines for Management of Type 2 Diabetes 2018. <https://medibulletin.com/wp-content/uploads/2018/05/ICMR.diabetesGuidelines.2018.pdf> (accessed May 2020).
      45. Seino Y, Nanjo K, Tajima N et al. (2010) Report of the Committee on the classiﬁcation and diagnostic criteria of diabetes mellitus. The Committee of the Japan Diabetes Society on the diagnostic criteria of diabetes mellitus. *Diabetol Int* **1**, 2–20.
      46. Singapore Ministry of Health. Diabetes Mellitus (Summary Booklet) (2014) MOH Clinical Practice Guidelines. <https://www.moh.gov.sg/docs/librariesprovider4/guidelines/cpg_diabetes-mellitus-summary-card---jul-2014.pdf> (accessed May 2020).
      47. [Ogbera](https://www.ncbi.nlm.nih.gov/pubmed/?term=Ogbera%20AO%5BAuthor%5D&cauthor=true&cauthor_uid=25512795) AO & [Ekpebegh](https://www.ncbi.nlm.nih.gov/pubmed/?term=Ekpebegh%20C%5BAuthor%5D&cauthor=true&cauthor_uid=25512795) C. (2014) Diabetes mellitus in Nigeria: The past, present and future. [*World J Diabetes*](https://www.ncbi.nlm.nih.gov/pmc/articles/PMC4265879/) **5**, 905–911.
      48. Diabetes South Africa (2017) Diabetes and Pregnancy. <https://www.diabetessa.org.za/diabetes-and-pregnancy/> (accessed May 2020).
      49. World Health Organization and International Diabetes Foundation (2006) Definition and diagnosis of diabetes mellitus and intermediate hyperglycemia. <https://apps.who.int/iris/bitstream/handle/10665/43588/9241594934_eng.pdf;jsessionid=13AC042BA8FA662E503F6062AB019274?sequence=1> (accessed May 2020).
      50. World Health Organization (2013) Diagnostic criteria and classification of Hyperglycaemia First Detected in Pregnancy. <https://www.paho.org/hq/dmdocuments/2015/guide-who-hyperglycaemia-pregnancy.pdf> (accessed May 2020).
      51. Pan American Health Organization. The U.S.–Mexico Border Diabetes Prevention and Control Project Phase I: Prevalence study of type 2 diabetes and its risk factors. <http://iris.paho.org/xmlui/bitstream/handle/123456789/3489/fep003173.pdf?sequence=1&isAllowed=y> (Accessed May 2020).
      52. [Aziz](javascript:;) A, [Dumais](javascript:;) L, [Barber](javascript:;) J. (2013) Health Canada’s evaluation of the use of glycemic index claims on food labels. *Am J Clin Nutr* **98**, 269-74.
      53. Diabetes Canada (2020) The Glycemic Index (GI). <https://www.diabetes.ca/managing-my-diabetes/tools---resources/the-glycemic-index-(gi)> (accessed May 2020.
      54. National Institute of Diabetes and Digestive and Kidney Disease. Eating, Diet, & Nutrition for NAFLD & NASH. <https://www.niddk.nih.gov/health-information/liver-disease/nafld-nash/eating-diet-nutrition> (accessed May 2020).
      55. American Diabetes Association. (2011) Position statement. Standards of Medical Care in Diabetes. *Diabetes Care* **34**, S11-S61.
      56. European Food Safety Authority (2010) Scientific Opinion on Dietary Reference Values for carbohydrates and dietary fibre*. EFSA Journal* **8**, 1462.
      57. Agence Francias de Securite Sanitaire des Aliments (2005) Glucides et santé : Etat des lieux, évaluation et recommandations. <http://www.anses.fr/sites/default/files/documents/NUT-Ra-Glucides.pdf> (accessed June 2020).
      58. Hauner H, Bechthold A, Boeing H et al. (2012) Evidence-based guideline of the German Nutrition Society: Carbohydrate intake and prevention of nutrition-related disease. *Ann Nutr Metab* **60**, S1-S-58.
      59. Public Health England Scientific Advisory Committee on Nutrition (2015). Carbohydrates and Health. <https://assets.publishing.service.gov.uk/government/uploads/system/uploads/attachment_data/file/445503/SACN_Carbohydrates_and_Health.pdf> (accessed May 2020).
      60. National Health Service (2018) What is Glycaemic Index (GI)? <https://www.nhs.uk/common-health-questions/food-and-diet/what-is-the-glycaemic-index-gi/> (accessed June 2020).
      61. Diabetes UK. Glycaemic index and diabetes. <https://www.diabetes.org.uk/guide-to-diabetes/enjoy-food/carbohydrates-and-diabetes/glycaemic-index-and-diabetes> (accessed May 2020).
      62. Food Safety Authority Australia New Zealand (2018). Getting Your Claims Right. <https://foodregulation.gov.au/internet/fr/publishing.nsf/Content/31BDC68CEC4A1964CA25801B00166C1F/$File/Getting-Your-Claims-Right-2018.pdf> (accessed May 2020).
      63. Food Safety Authority Australia New Zealand (2016) Nutrition, Health and Related Claims. <http://www.foodstandards.gov.au/industry/labelling/Pages/Nutrition-health-and-related-claims.aspx> (accessed May 2020).
      64. Australian government. [Australia New Zealand Food Standards Code – Standard 1.2.7 – Nutrition, health and related claims](https://www.legislation.gov.au/Details/F2015L00394/Download) . <https://www.legislation.gov.au/Details/F2015L00394/Download>. (accessed November 2020).
      65. Diabetes Australia (2020) Glycemic Index. <https://www.diabetesaustralia.com.au/glycemic-index> (accessed May 2020).
      66. Glycemic Index Foundation (2017). Making low GI General Level Health Claims. <https://www.gisymbol.com/wp-content/uploads/2017/08/GI-Foundation-Health-Claim-Presenter-June-2015.pdf> (accessed May 2020).
      67. Glycemic Index Foundation. What about glycemic load? <https://www.gisymbol.com/what-about-glycemic-load/> (accessed May 2020).
      68. Food Safety and Standards Authority of India (2018). Food Safety and Standards (Advertising and Claims) Regulations. <https://fssai.gov.in/upload/uploadfiles/files/Gazette_Notification_Advertising_Claims_27_11_2018.pdf> (accessed May 2020).
      69. Singapore Health Promotion Board (2019). A Handbook on Nutrition Labelling (Singapore). <https://www.hpb.gov.sg/docs/default-source/default-document-library/a-handbook-on-nutrition-labelling---revised-mar-2019.pdf?sfvrsn=7eb7c272_2> (accessed May 2020).
      70. South African Department of Health. (2014) Foodstuffs, Cosmetics and Disinfectants Act, 1972 (Act No. 54 of 1972). Regulations relating to the labelling and advertising of foods. <https://www.gov.za/sites/default/files/gcis_document/201409/37695rg10205gon429.pdf>. (accessed November 2020).
      71. Food Advisory Consumer Service (2019) Glycaemic Index. <https://foodfacts.org.za/glycaemic-index/> (accessed May 2020).
      72. The Glycemic Index Foundation of South Africa. <https://www.gifoundation.com/> (accessed May 2020).
      73. Mann J, Cummings JH, Englyst HN et al. (2007) FAO/WHO Scientific Update on carbohydrates in human nutrition: conclusions. *European Journal of Clinical Nutrition* **61**, S132–S137.
      74. Food and Agriculture Organization (1998). Carbohydrates in human nutrition. <http://www.fao.org/3/W8079E/w8079e07.htm#availability%20and%20consumption> (accessed May 2020).
