# Supplementary material for: Global evaluation of the use of glycaemic impact measurements to food or nutrient intake
Source: Public Health Nutr. 2021 Feb 10;24(12):3966–75. doi: 10.1017/S1368980021000616 (PMC8369455; doi:10.1017/S1368980021000616)
Supplement: Supplementary file 1 [file S1368980021000616sup.zip › S1368980021000616sup002.docx]

| **Supplemental Table 1. Post-prandial Glucose Area-Under-The-Curve (PPG AUC)** | | | | |
| --- | --- | --- | --- | --- |
| **Country/Region*** | **Regulatory Agency/Public Health/Ministry of Health**  **Scientific use**  **Cut-off/threshold**  **References on guidance, specific**  **conclusions/recommendations** | **Regulatory Use** | **Health (e.g., Diabetes) Associations**  **Scientific use/recommendations**  **Cut-off/threshold**  **References on guidance, specific**  **conclusions/recommendaitons** | **Association Use** |
| **Americas** | | | | |
| **Canada** | **Health Canada (1-3)**  For evaluating a function claim on reducing PPG, the primary outcome considered was the PPG response measured by determining blood glucose concentrations over at least a 2-h period after a test or control meal is consumed, and calculating the iAUC. The iAUC is the area over the baseline and under the glucose curve.  Measurements should be taken for an appropriate period of time. Generally, measurements should be taken for at least 2 hours, with higher frequency (for example, at 15-minute intervals) in the first hour, and 30 minutes thereafter.  In order to support a claim related to the reduction of glycaemic response, a minimum 20% decrease in the average incremental area under the glucose curve in comparison to the reference food is generally considered a physiologically relevant change (Health and Welfare Canada 1985). This magnitude of change must also be statistically significant. (1,2,3)    Novel fibers  Unclear if use PPG AUC or other PPG measurements | Function claim on postprandial glucose  Identification of novel fibers |  |  |
| **USA** | **Food and Drug Administration (FDA) (4,5)**  Reduced PPG AUC is a physiological effect of a non-digestible carbohydrate that is beneficial to human health. Beneficial effect that is used to meet the FDA dietary fiber definition for food labeling.  Statistically significant change in AUC based on P < 0.05 when compared to the control. | For evaluating the beneficial physiological effect (attenation of blood glucose) of non-digestible carbohydrates  Example: Identification of resistant starch 2 as a dietary fiber  Declaration of the amount of dietary fiber on the food label |  |  |
| **Europe** | | | | |
| **European Union** | **European Food Safety Authority (EFSA) (6)**  PPG AUC is an appropriate as primary endpoint to substantiate health claims regarding the reduction in PPG, whereas it can be used only as supportive evidence to substantiate health claims in the context of (long-term) maintenance of normal glucose regulation. However, it should be used in  combination with AUC insulin to exclude a disproportionate increase in insulin values in comparison with the control food/meal.  Less emphasis should be placed upon the reporting of statistical significance and more on statistical (point) estimation (e.g. of an effect) and associated interval estimation (e.g. confidence interval). It is considered that appreciably more information can be presented in the estimate of the size of an effect and its uncertainty when described by a confidence interval than when expressed solely by the results of significance tests. | Several function claims on post-prandial glycaemic impact |  |  |
| **Oceana** | | | | |
| **Australia**  **New Zealand** | **Food Standards Austrlaia New Zealand (FSANZ)(7)**    PPG AUC was not chosen as the measurement for postprandial blood glucose concentrations (see peak PPG). | Not used |  |  |
| **Asia** | | | | |
| **Middle East** | | | | |
| **Africa** | | | | |
| **International** | | | | |
|  |  |  | **IDF (8)**  The overall glucose exposure in a given individual can be estimated by calculating the total area under the 24-h glycaemic profile above zero. PPG is measured by calculating the AUC above the preprandial values over a 4-h period after the start of the meal. The choice of the 4-hour value is dictated by the mean duration of the hydrolysis and absorption of dietary carbohydrates (the so called postprandial state) which equals 4 hours both in normal individuals and in people with diabetes. |  |

| **Supplemental Table 2. Peak (or single time measure) post-prandial blood glucose** | | | | |
| --- | --- | --- | --- | --- |
| **Country/Region*** | **Regulatory Agency**  **Scientific use**  **Cut-off/threshold**  **References on guidance,**  **Specific conclusions/recommendations** | **Regulatory use** | **Health Association**  **Scientific use/recommendations**  **Cut-off/threshold**  **References on guidance, specific**  **conclusions/recommendaitons** | **Association use** |
| **Americas** | | | | |
| **Brazil** |  |  | **Brazil Diabetes Society (9)**  Recommended 2-h PPG <160 mg/dL  Acceptable 2-h PPG <130 mg/dL  Therapeutic 2-h PPG <180 mg/dL | Targets |
| **Canada** | **Health Canada (2)**  Peak level (highest level) and time to peak for blood glucose or insulin are not sufficient to measure response, but can be used as supportive data, when correlating with area under the curve. | Not used |  |  |
| **USA** | US Food & Drug Administration (4,5)  Peak PPG level is measured usually at 30 min after consumption  Reduced peak PPG is a physiological effect of a non-digestible carbohydrate that is beneficial to human health. Beneficial effect that is used to meet the FDA dietary fiber definition.  Statistically significant change in peak PPG based on P < 0.05 when compared to the control.  **Center for Disease Control and Prevention (10)**  Diabetes  2-h PPG < 180 mg/dL | For evaluating the beneficial physiological effect (attenation of blood glucose)  Declaration of the amount of dietary fiber on the food label  Identification of resistant starch 2 as a dietary fiber  Target | **American Diabetes Association (11-14)**  Fasting and postprandial self-monitoring of blood glucose are recommended in both gestational diabetes mellitus and preexisting diabetes in pregnancy to achieve glyacemic control.  Measuring PPG 1–2 h after the start of a meal and using treatments aimed at reducing PPG values to <180 mg/dL may help to lower A1C.  Gestational diabetes   - 1-h PPG <140 mg/dL - or - 2-h PPG <120 mg/dL | Clinical practice recommendations  Targets for most nonpregnant adults with diabetes. |
| **Europe** | | | | |
| **European Union** | **EFSA (15)**  Peak PPG is an appropriate primary endpoint to substantiate health claims regarding the reduction in PPG, whereas it can be used only as supportive evidence to substantiate health claims in the context of (long-term) maintenance of normal glucose regulation. However, it should be used in combination with peak plasma/serum insulin concentration to exclude disproportionate insulin values in comparison with the control food/meal. | Function claim for short-term reduction of PPG | **European Society of Cardiology/European Association for the Study of Diabetes (16)**  Type 2 diabetes  Peak PPG 135 mg/dL  Type 1 diabetes  Peak PPG 135-160 mg/dL | Targets |
| **United Kingdom** |  |  | **Diabetes UK (17)**  Diabetes  2-h PPG < 8.5mmol/L(155 mg/dL) | Target |
| **Oceana** | | | | |
| **Australia/New Zealand** | **FSANZ (18)**  Review on pectin/beta-glucan consumption and postprandial blood glucose concentrations. Peak postprandial blood glucose concentrations was chosen as the most appropriate measure of because this is the most uniformly reported measurement and also measures immediate postprandial effect. Selected the highest reported blood glucose concentration measurement after ingestion of a meal or glucose drink.    **New Zealand Ministry of Health (19)**  Gestational Diabetes  Monitor the blood glucose of women who have been diagnosed with gestational diabetes before breakfast (fasting blood sugar) and two hours after meals for 24 hours after delivery. Refer to the medical team if values are between 7 mmol/L and ≥ 11 mmol/L on two consecutive occasions.  Blood glucose treatment targets are:   - 1-h PPG ≤ 7.4 mmol/L - 2-h PPG ≤ 6.7mmol/L. | Review of health claims  Monitoring | **Diabetes Australia (20)**  T2D  2-h PPG 6-10 mmol/L (108-180 mg/dL)  T1D  2-h PPG < 10 mmol/L (180 mg/dL) | Target |
|  |  |  |  |  |
| **Fiji** | **Ministry of Health and Medical Services (21)**  Target during pregnancy  2-h PPG 5-7 mmol/L  Provide insulin if 2-h PPG > 7 mmol/L | Target and monitoring |  |  |
| **Asia** | | | | |
| **China** |  |  |  |  |
| **India** | **Minstry of Health and Family Welfare (22)**  2-h PPG should be maintained at a level < 120 mg/dL | Management of gestational diabetes |  |  |
| **Middle East** | | | | |
| **Pakistan** |  |  | **Diabetic Association of Pakistan (23)**  Diabetes  2-h PPG < 180 mg/dL | Target |
| **Africa** | | | | |
| **South Africa** |  |  | **Diabetes South Africa (24)**  Diabetes  PPG < 10 mmol/L (does not provide time) | Target |
| **International** | | | | |
|  |  |  | **International Diabetes Federation (25,26)**  Hyperglycemia  2- h PPG > 140mg/dL  Diabetes  Despite the postmeal plasma glucose levels being below 7.8 mmol/l (140 mg/dL) in people with normal glucose tolerance, glucose levels in healthy people are often difficult to achieve in people with diabetes without an undue risk of hypoglycaemia. Therefore, for reasons of safety, the IDF sets a glycaemic target slightly above the normal levels and for postmeal glucose this target is 9.0 mmol/L (160 mg/dL). | Targets |
|  |  |  | **Pan American Health Organization (PAHO) (27)**  **Below WHO information on Paho website** | Diagnosis |
|  |  |  | **World Health Organization (WHO) (28)**  A person who has diabetes is defined as someone with a postprandial (approximately 2 hours after a main meal) plasma glucose concentration above 11.0 mmol/L (200 mg/L) on two separate occasions) | Diagnosis |

| **Supplemental Table 3. Oral Glucose Tolerance Test (OGTT)** | | | | |
| --- | --- | --- | --- | --- |
| **Country/Region*** | **Regulatory Agency** | **Regulatory use** | **Health Association** | **Association Use** |
| **Americas** | | | | |
| **Brazil** |  |  | **Brazil Diabetes Society (9)**  To diagnose diabetes, two fasting plasma glucose values ≥ 126 mg/dL or a casual glycemia ≥ 200 mg/dL are necessary. After undergoing a 2-hour 75 g oral glucose tolerance test, the diagnostic criterion for diabetes is a plasma glucose ≥200 mg/dL. For impaired fasting glycemia, the diagnostic criteria are ≥100 mg/dL and <126 mg/dL, and for impaired glucose tolerance, the criterion is a 2-hour plasma glucose ≥140 mg/dL and <200 mg/dL  In 2014, the BDS reviewed its recommendations for glyacemic targets in the treatment of T2DM in order to make them more applicable and realistic. Glyacemic goals were defined as “recommended” (fasting and preprandial glycemia <100 mg/dL, and postprandial glycemia <160 mg/dL) or “acceptable” (fasting and preprandial glycemia <130 mg/dL and postprandial glycemia <180 mg/dL) for therapeutic adjustment. | Diagnosis and targets |
| **Canada** | **Public Health Canada (29)**  Dysglycemia, a positive OGTT, referred to an individual having a 2-h plasma glucose of ≥ 140 mg/dL | Canadian sub-population monitoring of diabetes risk | **Canadian Diabetes Association (30,31)**  Diabetes  2- h OGTT > 200 mg/dL  Prediabetes  2- h OGTT 140-200 mg/dL  Within 6 weeks to 6 months postpartum, with a 2 hour OGTT | Diagnosis  Screening |
| **USA** | **FDA (32)**  Surrogate endpoint for type 2 diabetes risk  OGTT test plasma glucose value of > 140 mg/dL and < 199 mg/dL is a risk factor for type 2 diabetes.  Significant change in OGTT based on P < 0.05 when compared to control.  **National Institutes of Health (33)**  Diagnosis of prediabetes or impaired glucose tolerance  2-h 140–199 mg/dL  Diagnosis of diabetes  2-h ≥ 200 mg/dL  **Center for Disease Control and Prevention (34)**  Screening of diabetes | Substantiate a health claim for type 2 diabetes  Diagnosis  NHANES Screening | **American Diabetes Association (35,36)**  Normal < 140 mg/dL  Prediabetes 140-199 mg/dL  Diabetes > 200 mg/dL  Gestational diabetes  1-h > 180 mg/dL  2-h > 155 mg/dL | Diagnosis |
| **Europe** | | | | |
| **European Union** | **EFSA (37)**  Function claim for glucose tolerance  Long-term increase in glucose tolerance is a beneficial physiological effect as long as serum insulin concentrations are not disproportionately increased. The scientific evidence for the substantiation of health claims related to an increase in glucose tolerance can be obtained from human intervention studies showing a decrease in blood glucose concentrations at different time points during a standard (WHO, 1999) OGTT and with no disproportionate increase in insulin concentrations following chronic consumption (at least 12 weeks) of the food that is the subject of the health claim. | Food Labeling |  |  |
| **United Kingdom** |  |  | **Diabetes UK (38)**  Impaired glucose tolerance  140 - 200 mg/dL  Diabetes  2-h ≥ 200 mg/dL | Diagnosis |
| **Oceana** | | | | |
| **Australia/New Zealand** | **Australia Government Department of Health – Health Direct (39)**  An oral glucose tolerance test (OGTT): The patient, who has already fasted, drinks a sugary drink and then has a blood test done, first one and then 2 hours later. Before the OGTT, the patient needs to eat and drink 150 grams of [carbohydrates](https://www.healthdirect.gov.au/carbohydrates) (found in starchy foods) each day for 3 days.  <https://www.healthdirect.gov.au/diabetes-diagnosis>  **FSANZ (40)**  Function claim for glucose tolerance  Long-term increase in glucose tolerance is a beneficial physiological effect as long as serum insulin concentrations are not disproportionately increased. The scientific evidence for the substantiation of health claims related to an increase in glucose tolerance can be obtained from human intervention studies showing a decrease in blood glucose concentrations at different time points during a standard (WHO, 1999) OGTT and with no disproportionate increase in insulin concentrations following chronic consumption (at least 12 weeks) of the food that is the subject of the health claim.  New Zealand Ministry of Health (19)  Early pregnancy  Women with an HbA1c 41–49 mmol/mol should be offered a two-hour, 75 g oral glucose tolerance test (OGTT) at 24–28 weeks.   - offer a1-h, 50 g oral glucose challenge test (polycose) for all women whose HbA1c is ≤ 40 mmol/mol | Guidance on testing | **Diabetes Australia (41)**  OGTT is performed six weeks after delivery to ensure that blood glucose levels have returned to normal. | Guidance on testing |
|  |  | Food Labeling  Guidance on testing | Diabetes New Zealand(42)  An oral glucose tolerance test is a test where you go to the laboratory before eating anything in the morning. A blood glucose level is taken. Then you will be asked to drink a glass of fluid containing 75 grams of glucose. The most accurate results of an OGTT will be achieved if it is done after you have had a diet with normal amounts of carbohydrate and moderate exercise over the previous three days. If you are not pregnant and your blood glucose two hours after an OGTT is 11.1 mmol/L or more you have diabetes.  All pregnant women in New Zealand are asked to have a “glucose challenge” test when they are 28 weeks pregnant. A glucose challenge test is exactly the same as an OGTT except the amount of glucose you are asked to drink is 50 grams not 75 grams.  If you are having a glucose tolerance test to see whether you have diabetes of pregnancy (gestational diabetes), and your blood glucose two hours after the glucose drink is 9mmol/L (162 mg/dL)or more, you have diabetes of pregnancy (gestational diabetes). | Guidance on testing |
| **Fiji** | **Fiji Ministry of Health and Medical Services(21)**  Inpaired glucose tolerance  Between 6.5 and 11 mmol/L (117-200 mg/dL)  Gestational Diabetes  1-h > 10 mmol/L  2-h > 8.5 mmol/L | Diagnosis |  |  |
| **Asia** | | | | |
| **China** |  |  | **Chinese Diabetes Society (43)**  Impaired glucose tolerance  ≥ 7.8 to <11.1 mmol/L  Diabetes  ≥ 11.1 mmol/L | Diagnosis |
| **India** | **Minstry of Health and Family Welfare (22)**  Gestational diabetes  OGTT ≥ 140 mg/dL  **Indian Council of Medical Research (44)**  2-h < 140 mg/dL (normal)  2-h 140-199 mg/dL (IGT)  2-h ≥ 200 mg/dL (diabetes) | Diagnosis of gestational diabetes  Diagnosis of diabetes |  |  |
| **Japan** |  |  | **Japanese Diabetes Society (45)**  Hyperglycemia  2-h > 200 mg/dL  Gestational diabetes  Diagnosed if one or more of the following criteria is met:  1-h > 180 mg/dL  2-h > 155 mg/dL | Diagnosis |
| **Singapore** | **Ministry of Health (46)**  For women with gestational diabetes, a 75 g 2-h OGTT should be performed 6–12 weeks postpartum and the woman reclassified and counselled according to criteria accepted in the non-pregnant state.  Gestational diabetes is diagnosed with a 75 g OGTT. A fasting venous plasma glucose ≥7.0 mmol/L or a 2-hour venous plasma glucose of ≥7.8 mmol/l is diagnostic of gestational diabetes. Casual venous plasma levels ≥11.1 mmol/L on 2 successive occasions would confirm gestational diabetes without recourse to oral glucose tolerance testing | Diagnosis |  |  |
| **Middle East** | | | | |
| **Africa** | | | | |
| **Nigeria** |  |  | **Diabetes Association of Nigeria (47)**  Recommends the performance of the 75 g OGTT in pregnant work with risk factors for gestational diabetes. | Guidance on testing |
| **South Africa** |  |  | **Diabetes South Africa (48)**  Diagnosis of gestational diabetes  1-h > 10 mmol/L  2-h > 8. 5mmol/L | Diagnosis |
| **International** | | | | |
|  |  |  | **WHO and IDF (49,50)**  An OGTT is the only means of identifying people with IGT. An OGTT is frequently needed to confirm or exclude an abnormality of glucose tolerance in asymptomatic people. An OGTT should be used in individuals with fasting plasma glucose of 110–125 mg/dL to determine glucose tolerance status.  Diabetes  2–h > 200 mg/dL  IGT  2-h > 140-200 mg/dL | Diagnosis |
|  |  |  | **PAHO (51)**  Individuals with impaired glucose tolerance of 140–199 mg/dL (7.8–11.0 mmol/L) are associated with increased cardiovascular disease and could progress to T2DM, especially if other risk factors exist. | Risk of T2D |

| **Supplemental Table 4. Glycaemic Index (GI) and Glycaemic Load (GL)** | | | | |
| --- | --- | --- | --- | --- |
| **Country/Region*** | **Regulatory agency** | **Regulatory Use** | **Health Associations** | **Association Use** |
| **Americas** | | | | |
| **Canada** | **Health Canada (52)**  Inclusion of the GI value on the label of eligible food products would be misleading and would not add value to nutrition labeling and dietary guidelines in assisting  consumers to make healthier food choices. | Not Used | **Diabetes Canada (53)**  For individuals with pre-diabetes or diabetes, Diabetes Canada recommends choosing lower GI foods and drinks more often to help control blood sugar.  Diabetes Canada recommends that Canadians living with diabetes choose lower GI foods and drinks most often to help control blood sugar.  Diabetes Canada’s most recent education materials have been designed to support healthcare providers and people affected by diabetes as they learn about GI together. | Guidance and Education on food choices and meal planning |
| **USA** | **FDA (4)**  GI does not measure physiological benefits of nutrients added to foods such as dietary fiber.  **National Institutes of Health (54)**  For nonalcoholic fatty liver disease or nonalcoholic steatohepatitis:  Eat more low-GI foods—such as most fruits, vegetables, and whole grains. These foods affect your [blood glucose](https://www.niddk.nih.gov/Dictionary/B/blood-glucose) less than high-glycemic index foods, such as white bread, white rice, and potatoes. | Not used | American Diabetes Association (55)  While recommendations are not provided, based on the American Diabetes Association evidence grading system for clinical practice recommendations, it was concluded that there was “supporting” evidence from observational studies that the use of the glycemic index and glycemic load may provide a modest additional benefit for glycemic control over that observed when total carbohydrate is considered alone. | None |
| **Europe** | | | | |
| **European Union** | **EFSA (56)**  The incremental area under the blood glucose response curve following a 50 g glycaemic carbohydrate portion of a test food expressed as a percent of the response to the same amount of carbohydrates from a standard reference product taken by the same subject (FAO/WHO, 1998).  A cause-effect relation could not be  established between low-GI carbohydrate foods and the claimed functional effects.  Although there is some experimental evidence that a reduction of the dietary GI and GL may have favourable effects on some metabolic risk factors such as serum lipids, the evidence for a role in weight maintenance and prevention of diet-related diseases is inconclusive. | Not used |  |  |
| **France** | **L’Agence française de sécurité sanitaire des aliments (Afssa) (57)**  Currently level of evidence of interest in low GI foods is not enough to make it  health recommendations for the general population  The GI remains a useful parameter for diabetic subjects and facilitating their food choices, but should also consider the overall composition of their diet.  (Translated/Not in English) | Dietary Guidance |  |  |
| **Germany** |  |  | **German Nutrition Society (58)**  The recently issued German Nutrition Society DRV document reports that: “to date there is only possible evidence regarding a risk-increasing effect of high Glycaemic Index on some nutrition-related diseases. Therefore, no recommendations are made in that respect.” | Not used |
| **United Kingdom** | **Public Health England (59)**  **Scientific Advisory Committee on Nutrition**  It is not possible to assign cause-effect relationships for outcomes based on variation in diet GI or GL, as higher or lower GI diets differ in many ways other than just the carbohydrate fraction. | Not used | **Diabetes UK (60)**  - Research has shown that choosing Low GI foods can particularly help manage glucose levels in people with Type 2 diabetes. There is less evidence to suggest it can help with blood glucose control in people with Type 1 diabetes.  - Not all low-GI foods are healthy choices – chocolate, for example, has a low-GI because of its fat content, which slows down the absorption of carbohydrate.  -Combining foods with different GIs alters the overall GI of a meal. You can maximize the benefit of GI by switching to a low GI option with each meal or snack. Go easy on lower GI foods like chocolate, which is high in fat and calories, especially if you are trying to lose weight. Save them for occasional treats.  - Eating to control your diabetes isn’t just about GI ratings. Think of the bigger picture and choose foods low in saturated fat, salt and sugar as part of a healthy, balanced diet. | General food guidance |
| **Oceana** | | | | |
| **Australia/New Zealand** | **FSANZ (61-64)**  FSANZ allows companies to make nutrient content claims and use front-of-package labeleing regarding the GI of a food.Nutrition content claims can be made about GI and GL but the food must meet the Nutrient Profiling Score Criterion. When making a claim about GI the specific numerical value of the GI of the food must be included either in the claim or in the nutrition information panel. The descriptors low, medium and high are optional in a GI claim but if used must meet the certain conditions  Low GI < 55  Medium GI 56-69  High > 70  When making a claim about GL only a number or a descriptor in numeric form can be used. | Nutrient content and function claims  Front of Package labeling | **Diabetes Australia (65)**  The recommendation is to eat more low and intermediate GI foods, not to exclude high GI foods.  **Glycemic Index Foundation (66,67)**  The GI symbol**, G – GI tested,** is an endorsement program which indicates the GI rating of packaged food products in supermarkets. It ranks food products based on the speed at which they break down from carbohydrate to sugar in the bloodstream. However, this labelling is not compulsory for food companies to follow.  The GI symbol is a front-of-pack labeling scheme that also includes the requirement to include a GI value in the Nutrition Facts/Nutrition Information Panel.  The GI symbol only appears on food products that meet certain nutrient criteria for that food category. High and intermediate GI soft drinks, cordials, syrups, confectionery and sugars are excluded. Jams, honey and other carbohydrate-containing spreads are not necessarily excluded. | Food Labeling/GI Symbol/ Function claims  Dietary guidance |
| **Fiji** | **Fiji Ministry of Health and Medical Services (21)**  Individuals with diabetes aim to consume foods with a low GI. Not defined. Whole meal products and leafy vegetables | Guidance/management of diabetes |  |  |
| **Asia** | | | | |
| **India** | **Food Safety and Standards Authority of India (68)**  Low GI:  GI value below 55 A food’s GI indicates the rate at which the carbohydrate in the food is broken down into glucose and absorbed from the gut into the blood and expressed as a per cent of the response to the same amount of carbohydrate from a standard food, white bread  **Ministry of Health and Family Welfare (22)**  The total intake of carbohydrate should be controlled and monitored and carbohydrate  foods with a lower GI should be emphasized.  **Indian Council of Medical Research (44)**  It is recommended that carbohydrates from foods high in fi bre e.g. whole grains (unpolished cereals and millets), legumes, peas, beans, oats, barley and some fruits with low glycaemic index and glycaemic load are consumed. | Food labeling- nutrient content claims  Dietary Guidance with diabetes |  |  |
| **Singapore** | **Health Promotion Board (69)**  GI testing should be performed using an in-vivo GI testing according to the SAC-SINGLAS Technical Notes FFT01-General criteria for testing of Health Related Properties of Food supported ISO 26642:2010(E) at an accredited laboratory.  For reference, the GI classification is Low: ≤ 55 ; Medium : 56 – 69 ; High :≥ 70  Allowed “Low Glycemic Index” claim:  Foods claiming to have Low Glycemic Index must have a GI value* of 55 and below.  The cut-off values for the classification of low, medium and high GI are standardised internationally (ISO 26642:2010). | Food labeling |  |  |
| **Africa** | | | | |
| **South Africa** | **South African Department of Health (70)**  For health claims, the GI category claim shall, if used, be indicated as either category "Low", "Intermediate" or "High", whatever is applicable, as determined in accordance with the International standard method for GI testing, ISO 26642 and shall not include any method whereby a glycaemic index value is calculated to determine its category.  Low GI Value: 0 to 55  Intermediate GI value: 56 to 69  High GI value: 70 and more  Voluntary GI information can be provided on labels by way of the GI Foundation of South Africa (GIFSA) endorsement logo which has been accredited by the Dept of Health, as the GI testing done by them is in accordance with international standards and must meet other criteria. The food labelling regulations make no other provision for other GI information on a food label to date.  A GL claim is permissible only if the GI category is indicated as well. | Food Labeling | **Food Advisory Consumer Service (71)**  GI value can assist in selecting food that is high in fibre, micronutrients and antioxidants and low in energy – which is the basis of a healthy diet.  Carbohydrate containing foods according to the effect they have on blood glucose levels after they have been eaten and digested. In other words, it is a measure of the rate at which blood glucose levels are increased after eating a carbohydrate food, such as sugar or a carbohydrate containing food, such as bread.  **GI Foundation of South Africa (72)**  Symbol program | Dietary Guidance  Symbol for food labeling |
| **Middle East** | | | | |
| **International** | | | | |
|  |  |  | **WHO/Food and Agriculture Organization (FAO) (73)**  The choice of carbohydrate-containing foods should not be based solely on GI since low-GI foods may be energy dense and contain  substantial amounts of sugars, fat or undesirable fatty acids that contribute to the diminished glycaemic response but not necessarily to good health outcomes. The inter-individual variation in glycaemic responses to foods is a further limitation of the GI concept. GI is perhaps most appropriately used to guide food choices when considering similar carbohydrate-containing  foods. GI should always be considered in the context of other nutritional indicators.  While foods with a low GI may also confer benefit in some of these contexts, the scientific update suggests caution regarding the use of the GI as the sole determinant of the quality of carbohydrate-containing foods.  GL should always be considered in the context of other nutritional indicators.  **FAO (74)**  GI can be used, in conjunction with information about food composition, to guide food choices. For practical application, the glycaemic index is useful to rank foods by developing exchange lists of categories of low glycaemic index foods, such as legumes, pearled barley, lightly refined grains (e.g. whole grain pumpernickel bread, or breads made from coarse flour), pasta, etc. | Conclusions regarding quality of foods |
| *Information on all of the various measures of glycaemic impact was not found for the following countries: Argentina, Egypt, Ethiopia, Indonesia, Iran, Mexico, Papua New Guinea, Russian Federation, Solomon , South Korea, Spain, Taiwan, Tanzania, Thailand, Turkey, Ukraine, and Vietnam. | | | | |
